# 乳酸代谢重编程在非小细胞肺癌中的研究进展

**DOI:** 10.3779/j.issn.1009-3419.2026.106.03

**Published:** 2026-02-20

**Authors:** Jun ZHOU, Liuling GE, Jingting JIANG

**Affiliations:** ^1^213003 常州，苏州大学附属第三医院呼吸与危重症医学科; ^1^Department of Respiratory Medicine, The Third Affiliated Hospital of Soochow University, Changzhou 213003, China; ^2^213003 常州，苏州大学附属第三医院肿瘤生物诊疗中心; ^2^Department of Tumor Biological Treatment, The Third Affiliated Hospital of Soochow University, Changzhou 213003, China; ^3^213003 常州，江苏省肿瘤免疫治疗工程技术研究中心; ^3^Jiangsu Engineering Research Center for TumorImmunotherapy, The Third Affiliated Hospital of Soochow University, Changzhou 213003, China; ^4^213003 常州，苏州大学附属第三医院细胞治疗研究院; ^4^Institute of Cell Therapy, The Third Affiliated Hospital of Soochow University, Changzhou 213003, China

**Keywords:** 肺肿瘤, 乳酸代谢, 肿瘤微环境, 表观遗传, Lung neoplasms, Lactate metabolism, Tumor microenvironment, Epigenetics

## Abstract

非小细胞肺癌（non-small cell lung cancer, NSCLC）具有高发病率与高死亡率的临床特点，5年生存率较低。乳酸代谢在NSCLC代谢重编程中发挥核心作用。乳酸不仅作为糖酵解终产物在肿瘤微环境（tumor microenvironment, TME）中堆积并形成酸性环境，还能进入三羧酸循环参与能量代谢。此外，G蛋白偶联受体81（G protein-coupled receptor 81, GPR81）/磷脂酰肌醇3-激酶（phosphoinositide 3-kinase, PI3K）/哺乳动物雷帕霉素靶蛋白（mammalian target of rapamycin, mTOR）信号通路，诱导程序性死亡配体1、细胞毒性T淋巴细胞相关蛋白4等免疫检查点分子表达，抑制T淋巴细胞和自然杀伤细胞功能，形成免疫抑制微环境。乳酸促进上皮-间质转化和肿瘤转移，并通过组蛋白乳酸化修饰推动NSCLC耐药及复发。临床研究显示，乳酸代谢增强与NSCLC恶性进展和化疗耐药有关，靶向乳酸代谢与免疫治疗联合应用可协同抑瘤。因此，综合阻断乳酸代谢、增强免疫应答有望提升NSCLC精准治疗效果。

## Abstract (en)

Non-small cell lung cancer (NSCLC) is characterized by high incidence and mortality, with a low five-year survival rate. Lactate metabolism plays a central role in the metabolic reprogramming of NSCLC. Beyond serving as the end-product of glycolysis, lactate accumulates in the tumor microenvironment (TME), contributing to acidification, and can also enter the tricarboxylic acid cycle to participate in energy metabolism. Moreover, the G protein-coupled receptor 81 (GPR81)/phosphoinositide 3-kinase (PI3K)/mammalian target of rapamycin (mTOR) signaling axis induces the expression of immune checkpoint molecules, such as programmed death-ligand 1 and cytotoxic T lymphocyte-associated protein 4 (CTLA-4), thereby suppressing the functions of T lymphocytes and natural killer cells and establishing an immunosuppressive microenvironment. Lactate further promotes epithelial-mesenchymal transition and tumor metastasis, and drives NSCLC chemoresistance and relapse via histone lactylation. Clinical studies indicate that enhanced lactate metabolism is associated with NSCLC progression and chemotherapy resistance, while targeting lactate metabolism in combination with immunotherapy exerts synergistic antitumor effects. Therefore, comprehensive inhibition of lactate metabolism together with enhancement of antitumor immunity may improve the efficacy of precision therapy in NSCLC.

肺癌引起的死亡事件在全球癌症相关死亡中居首位，约占全部癌症死亡的18.7%^[1]^。其中，非小细胞肺癌（non-small cell lung cancer, NSCLC）作为主要的组织学亚型，约占肺癌总病例的85%^[2]^。针对NSCLC患者表皮生长因子受体（epidermal growth factor receptor, *EGFR*）、间变性淋巴瘤激酶（anaplastic lymphoma kinase, *ALK*）、ROS原癌基因1受体酪氨酸激酶（ROS proto-oncogene 1 receptor tyrosine kinase, *ROS1*）等突变基因的靶向药物和免疫治疗药物已在临床上广泛应用，显著改善了患者的生存预后，但受肿瘤异质性及耐药机制的限制，晚期患者的5年生存率仍低于20%^[3]^。肿瘤精准治疗进入快速发展阶段，但疗效的可持续性和广泛性仍面临挑战，探索新靶点成为NSCLC治疗的重要研究方向。

代谢重编程是肿瘤的核心特征之一，肿瘤细胞通过重塑代谢满足自身快速增殖的需求，增强迁移和侵袭能力^[4]^。有氧条件下肿瘤通过“Warburg效应”进行能量代谢，产生大量乳酸^[5]^。研究^[6]^表明，乳酸不仅是糖酵解的代谢终产物，还可作为信号分子调控细胞功能，参与免疫应答等多种生物过程。此外，NSCLC细胞糖酵解关键酶活性显著增强，激活下游多条信号通路，并上调乳酸转运体和相应感受器的表达水平，共同构建乳酸代谢重编程的调控网络^[7]^。有研究^[8]^证实乳酸在NSCLC免疫微环境塑造中发挥关键作用，其通过抑制效应免疫细胞的活性和激活免疫抑制性细胞来维持免疫抑制微环境。“免疫代谢重编程”不仅有利于NSCLC细胞规避宿主免疫监视，还可影响其免疫治疗效果。乳酸作为代谢与信号调控因子，在NSCLC的演进过程、代谢重编程、肿瘤微环境（tumor microenvironment, TME）调控以及免疫逃逸中发挥重要作用。本文旨在探讨NSCLC乳酸代谢重编程的功能机制及药物转化研究进展，为肿瘤治疗提供新思路。

## 1 乳酸驱动NSCLC恶性演进过程

### 1.1 乳酸促进NSCLC细胞增殖与存活

NSCLC细胞可直接摄取外源乳酸作为代谢燃料，促进肿瘤细胞增殖与生存。NSCLC患者体内存在“乳酸循环”，肿瘤核心区域产生乳酸，边缘区域则摄取和利用，有助于肿瘤细胞在低氧或营养限制条件下维持生存和增殖^[9]^。体外试验^[10]^表明，在Kirsten大鼠肉瘤病毒癌基因同源物（Kirsten rat sarcoma viral oncogene homolog, *KRAS*）突变型NSCLC细胞系中去除谷氨酰胺后添加乳酸，可显著增强肿瘤细胞的增殖与克隆形成能力。此外，乳酸可通过多种机制参与NSCLC细胞的糖代谢，并介导负责乳酸转运的单羧酸转运体1（monocarboxylate transporter 1, MCT1）表达上调，从而维持细胞内的酸碱平衡^[11]^。Zhang等^[12]^的研究证实乳酸通过促进组蛋白赖氨酸乳酸化修饰调控关键基因转录，发现乳酸上调组蛋白H3第18位赖氨酸乳酸化（histone H3 lysine 18 lactylation, H3K18la）水平，增强骨髓细胞瘤癌基因蛋白（MYC proto-oncogene protein, MYC）的活性和核转运，促进NSCLC细胞增殖。

### 1.2 乳酸驱动NSCLC细胞迁移和侵袭

乳酸可通过多条信号通路重塑NSCLC细胞外基质，从而促进细胞的迁移与侵袭能力。乳酸可激活转录因子Snail诱导上皮-间质转化（epithelial-mesenchymal transition, EMT）从而增强肿瘤细胞迁移能力，研究^[13]^发现乳酸通过激活Snail/转化生长因子β（transforming growth factor-β, TGF-β）复合体，重塑细胞外基质，促进肿瘤细胞迁移与侵袭。此外，乳酸通过增加活性氧（reactive oxygen species, ROS）和CD38表达，诱导Snail依赖性EMT，驱动NSCLC细胞迁移^[14]^。同时，NSCLC细胞中乳酸激活磷脂酰肌醇3-激酶/蛋白激酶B（phosphoinositide 3-kinase/protein kinase B, PI3K/AKT）通路并上调基质金属蛋白酶-2和基质金属蛋白酶-9，显著增强细胞迁移能力^[15]^。此外，乳酸可通过乳酸化修饰调控NSCLC细胞迁移能力，低氧通过诱导SRY盒转录因子9（SRY-box transcription factor 9, SOX9）乳酸化增强肿瘤细胞迁移能力^[16]^。另一项研究^[17]^发现，葡萄糖转运蛋白5（glucose transporter 5, GLUT5）介导的果糖代谢增强乳酸生成，导致磷酸化AKT上调，从而促进NSCLC细胞迁移，表明乳酸代谢重编程通过不同途径蓄积乳酸和激活信号通路促进肿瘤细胞迁移。

## 2 乳酸对NSCLC代谢重编程和TME调控的影响

### 2.1 乳酸介导NSCLC代谢重编程

乳酸调控NSCLC细胞代谢重编程，满足肿瘤细胞高增殖、高能量需求，并使其适应酸性微环境的代谢压力。NSCLC细胞高表达乳酸脱氢酶A（lactate dehydrogenase A, LDHA），促进丙酮酸转化为乳酸，并通过MCTs将乳酸外排或摄取以维持细胞内稳态^[18]^。缺氧诱导因子-1α（hypoxia-inducible factor 1alpha, HIF-1α）介导MCT4表达显著上调，形成以乳酸为核心的代谢环路，诱导NSCLC细胞向高侵袭性表型转化^[19]^。此外，乳酸可介导代谢重编程调控NSCLC的表观遗传修饰水平。乳酸通过上调NSCLC细胞H3K18la水平，激活代谢信号通路相关蛋白，增强其糖酵解活性^[20]^。乳酸诱导RNA结合蛋白15与METTL3-WTAP复合物结合增强整体m^6^A甲基化水平，维持m^6^A修饰介导的代谢基因转录稳定性^[21]^。同时，研究^[22]^还发现乳酸化修饰与代谢重编程之间存在交互机制，组蛋白乳酸化修饰激活m^6^A识别蛋白YTHDF2表达，促进NSCLC细胞下游糖酵解通路活性及肿瘤干性。乳酸诱导非代谢相关蛋白MOESIN发生乳酸化修饰，增强NSCLC细胞对其TME的适应性^[8]^。

### 2.2 乳酸调控NSCLC TME稳态与代谢通讯

TME是由肿瘤细胞、成纤维细胞、血管内皮细胞及细胞外基质等多种成分共同构建的动态生态系统。乳酸广泛参与TME内不同细胞群之间的代谢互作与物质交换，是调控TME稳态的重要介质^[23]^。乳酸可调节TME中pH值，致使NSCLC细胞乳酸外排形成局部酸化环境，并通过酸敏感受体GPR132调节成纤维细胞和血管内皮细胞功能，影响肿瘤血管灌注和通透性^[24]^。酸中毒TME重塑细胞外基质，促进血管生成，增强NSCLC细胞的转移与定植能力，促进肿瘤细胞的干性，提高其侵袭潜能和治疗耐受性^[25]^。

乳酸通过调控TME代谢重编程，从而影响细胞间代谢物交换。乳酸可由肿瘤相关成纤维细胞（cancer-associated fibroblasts, CAFs）产生，并通过反向“Warburg效应”刺激NSCLC细胞氧化磷酸化上调，为肿瘤细胞提供能量^[26]^。乳酸可诱导正常成纤维细胞糖代谢从线粒体氧化磷酸化转向糖酵解^[27]^。NSCLC中CAFs主要代谢方式也转向糖酵解，伴随TME纤维化和免疫抑制浸润，提示乳酸可能介导CAFs代谢重编程加速NSCLC进展^[28]^。Luo等^[29]^发现乳酸可重塑肺癌TME中细胞通讯网络，通过介导组蛋白乳酸化修饰调控胞外囊泡中miRNA和代谢酶表达，进而影响邻近细胞的代谢表型和信号传递。

## 3 乳酸代谢重编程介导的NSCLC免疫逃逸机制

代谢重编程是肿瘤免疫逃逸的关键机制之一。“Warburg效应”为肿瘤细胞提供更高效的能量利用，导致TME乳酸积累，形成酸性TME。这种环境不仅促进肿瘤细胞增殖和迁移，还抑制效应免疫细胞的功能，增强抑制性免疫细胞活性，诱导免疫抑制微环境，有利于肿瘤细胞免疫逃逸（图1）。下文将分别探讨乳酸对抑制免疫细胞功能、促进免疫抑制性细胞富集及调控免疫检查点表达的影响。

**图1:**
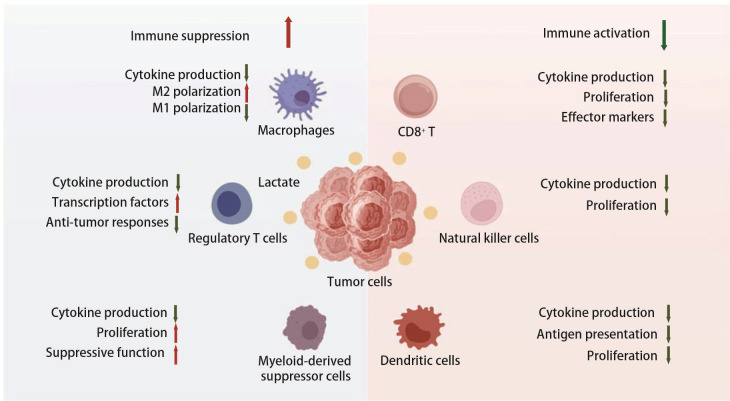
乳酸对免疫细胞的作用

### 3.1 乳酸抑制效应性免疫细胞功能

效应性免疫细胞具备直接识别并清除肿瘤细胞的能力，是抗肿瘤免疫反应的核心执行者，主要包括CD8⁺ T细胞、自然杀伤（natural killer, NK）细胞和树突状细胞（dendritic cell, DC）。乳酸积累导致NSCLC中TME酸化，进而重塑肿瘤细胞代谢，并显著抑制CD8⁺ T细胞、NK细胞、DC等关键效应免疫细胞功能，形成高度免疫抑制性TME，从而促进肿瘤的持续增殖和转移^[30]^。

CD8⁺ T细胞作为抗肿瘤免疫反应的主要细胞类型，在乳酸蓄积环境中功能受限。研究^[31]^表明，乳酸介导的线粒体应激诱导CD8⁺ T细胞代谢重编程，抑制其关键细胞毒性效应分子颗粒酶B和γ干扰素（interferon-gamma, IFN-γ）表达，从而削弱细胞增殖能力与杀伤活性，诱导其功能衰竭。多组学分析^[32]^显示，NSCLC组织中糖酵解酶LDHA的高表达与免疫相关基因呈负相关，尤其与CD8⁺ T细胞的浸润水平呈现显著负相关，提示乳酸代谢可能通过降低T细胞浸润从而促进肿瘤的侵袭性。

NK细胞对NSCLC细胞的免疫清除能力和免疫毒性同样受到乳酸抑制。乳酸可阻断活化T细胞核因子（nuclear factor of activated T-cells, NFAT）的核转位，抑制NK细胞的活化及IFN-γ的分泌，抑制其效应功能^[33]^。临床研究^[34]^进一步表明，NSCLC组织中m^6^A修饰相关基因表达升高，与糖酵解活性及NK细胞、B淋巴细胞的标志物表达呈显著负相关，提示m^6^A修饰可能通过调控代谢重编程抑制NK细胞浸润与功能。

DC在介导肿瘤抗原呈递和激活CD8⁺ T细胞中发挥关键作用，而肿瘤来源的乳酸可显著抑制DC的免疫功能。在NSCLC TME中乳酸抑制DC的功能，降低其促炎细胞因子白细胞介素（interleukin, IL）-12和IFN-γ的表达水平，从而削弱对初始CD8⁺ T细胞的激活能力^[35]^。

### 3.2 乳酸促进免疫抑制细胞的富集与功能维持

多种免疫抑制性细胞如调节性T细胞（regulatory T cells, Tregs）、髓源性抑制性细胞（myeloid-derived suppressor cells, MDSC）及肿瘤相关巨噬细胞（tumor-associated macrophage, TAM）等通过分泌免疫抑制因子或细胞接触，降低效应T细胞活性，形成免疫耐受环境。乳酸可激活免疫抑制细胞的免疫调节能力，重塑免疫抑制性TME，从而促进肿瘤的免疫逃逸。乳酸可通过多条信号通路增强Tregs、MDSC及TAM的免疫抑制功能，削弱免疫应答，影响免疫治疗的疗效。

Tregs在NSCLC中富集和功能增强，维持TME免疫抑制状态，削弱抗肿瘤免疫反应。Tregs中转录因子FOXP3高表达，驱动其免疫抑制表型的维持^[36]^。circRUNX1/miR-145/HK2信号轴通过调节“Warburg效应”促进肺癌组织中Tregs的代谢活性和增殖能力，进而增强其浸润能力^[37]^。此外，乳酸通过上调Tregs表面的免疫检查点分子细胞毒性T淋巴细胞相关抗原4（cytotoxic T-lymphocyte-associated protein 4, CTLA-4）的表达，并诱导其组蛋白乳酸化修饰，使Tregs在高乳酸、低葡萄糖的环境中维持功能稳定性，从而持续发挥免疫抑制作用^[38]^。

MDSC作为另一类重要的免疫抑制效应细胞，在NSCLC中大量浸润，形成免疫抑制TME。研究^[39]^显示，NSCLC组织中IL-6/JAK/STAT3通路激活，与MDSC的富集程度及患者不良预后密切关联。乳酸亦可通过调控Notch信号通路，影响MDSC的分化状态和功能。研究^[40]^表明Notch信号激活可减少乳酸积累，从而抑制MDSC活化及其免疫抑制效应。

M2型TAM是NSCLC免疫逃逸的关键细胞亚群之一。NSCLC细胞通过激活PI3K/AKT/哺乳动物雷帕霉素靶蛋白（mammalian target of rapamycin, mTOR）、Notch等通路上调C-C基序趋化因子配体2（C-C motif chemokine ligand 2, CCL2）、IL-1β等细胞因子，促进TAM向M2极化并增强其免疫抑制活性^[41]^。乳酸可通过激活GPR132/cAMP/PKA信号轴促进M2型TAM分化，增强其Arg1与IL-10等抑制性因子的表达^[42]^。此外，在IL-4诱导的M2型极化过程中乳酸作为三羧酸循环底物，通过三磷酸腺苷（adenosine triphosphate, ATP）柠檬酸裂解酶生成乙酰辅酶A，促进组蛋白乙酰化，从而驱动TAM向M2型极化并上调相关标志基因的表达^[43]^。

### 3.3 乳酸调控免疫检查点表达

免疫检查点在肿瘤细胞上异常激活导致免疫紊乱，有利于肿瘤细胞逃逸。乳酸代谢可通过影响免疫检查点分子的表达参与NSCLC免疫逃逸过程，影响免疫治疗的敏感性和疗效。

其他实体瘤模型中发现乳酸水平升高与免疫抑制细胞表面免疫检查点程序性死亡配体1（programmed death-ligand 1, PD-L1）和CD8⁺ T细胞表面程序性死亡受体1（programmed cell death protein 1, PD-1）、CTLA-4表达上调密切关联，从而维持广泛而持久的免疫抑制状态^[44,45]^。Feng等^[46]^发现乳酸可通过上调NSCLC细胞GPR81的表达，活化PD-L1启动子，上调PD-L1表达水平，进而抑制CD8⁺ T细胞活性和免疫杀伤功能。同时，临床研究^[47]^证实NSCLC患者中高乳酸水平与抗PD-1疗法疗效下降密切关联，提示乳酸代谢状态可作为免疫治疗反应的潜在预测指标。此外，乳酸通过诱导载脂蛋白C2第70位赖氨酸发生乳酸化，促进Tregs招募并增强PD-1抑制通路活性，导致NSCLC患者临床免疫治疗产生耐药性^[48]^。

## 4 乳酸代谢关键靶点在NSCLC治疗中的临床转化

### 4.1 乳酸代谢靶点治疗

临床部分NSCLC患者对放疗、化疗和免疫治疗等一线治疗产生耐药，迫切需要探索新的分子机制与靶点以改善疗效。乳酸代谢在NSCLC中高度活跃，抑制糖酵解相关酶、乳酸转运体和受体可阻断乳酸发挥作用，抑制肿瘤发生发展。研究^[49]^发现糖酵解的关键酶LDHA在NSCLC中高表达。然而，实体瘤动物模型研究^[50-52]^中发现LDHA抑制剂GNE-140、FX11等可以抑制肿瘤增殖，其竞争性抑制剂Machilin A不仅抑制NSCLC细胞的LDHA表达，还可诱导巨噬细胞向M2型分化和抑制血管生成，减弱肿瘤细胞转移能力。当使用乳酸转运体MCT1抑制剂时，可反向调节糖酵解逆转NSCLC的耐药现象^[53]^。此外，研究^[11]^发现GPR81是肿瘤细胞存活的关键受体，在肺癌中高表达，靶向GPR81和MCT1为表皮生长因子受体-酪氨酸激酶抑制剂（epidermal growth factor receptor-tyrosine kinase inhibitors, EGFR-TKIs）耐药肺癌提供了新思路。

目前，靶向乳酸代谢的药物已从基础研究逐渐步入临床探索阶段。研发药物对乳酸合成代谢和乳酸转运的关键靶点进行阻滞（表1^[50,51,54-70]^），其中主要以MCT1和MCT4为主要靶点。MCT1针对性小分子抑制剂AZD3965已经进入小细胞肺癌I/II期临床研究，抑制乳酸转运，发挥抗肿瘤作用，可作为监测的重要药效学指标之一^[54]^。同时，AZD0095作为一种可能用于临床的MCT4抑制剂，可直接提高胞内乳酸、抑制肿瘤细胞增殖，表现出明显的转化潜力^[55]^。

**表1:** 乳酸代谢相关靶点及药物

Mode of action	Mechanism of action	Target	Drug name	Research progress	Reference
Lactate transport inhibition	Inhibition of cellular uptake	MCT1	AZD3965	Phase I/II clinical trial	^[54]^
	Inhibition of cellular efflux	MCT4	AZD0095	Preclinical research	^[55]^
Lactate anabolism inhibition	Inhibition of glucose transport	GLUTs	Fasentin STF-31 WZB117 BAY-876	Preclinical research	^[56]^
			Silybin Ritonavir Genistein Apigenin	Phase I/II clinical trial	^[57,58]^
	Inhibition of glycolysis-related enzymes	HK2	microRNA lncRNA Kavain C	Preclinical research	^[59-61]^
			Lonidamine 2-DG	Phase I/II clinical trial	^[62,63]^
		PK	Shikonin Mannose Sanguinarine	Preclinical research	^[64-66]^
		LDH	FX11 GNE-140 Stiripentol	Preclinical research	^[50,51,67]^
			AT-101 Polyphenon E Sirpiglenastat	Phase I/II clinical trial	^[68-70]^

GLUTs: glucose transporters; HK2: hexokinase 2; PK: pyruvate kinase; LDH: lactate dehydrogenase; MCT1: monocarboxylate transporter 1; 2-DG: 2-deoxy-D-glucose; MCT4: monocarboxylate transporter 4.

### 4.2 乳酸代谢靶点联合治疗策略

多项研究证实靶向乳酸代谢可增强传统治疗和免疫治疗的效果，为NSCLC的药物研究指明新方向。Fan等^[71]^的研究发现顺铂作为化疗一线药物易产生耐药性，LDHA抑制剂或GLUT抑制剂可以抑制乳酸生成和细胞外乳酸堆积，从而降低顺铂对NSCLC细胞毒性及提高治疗敏感性；同时研究^[72]^结果表明代谢干预可协同顺铂诱导线粒体损伤、ROS释放与细胞凋亡，从而呈现更强的抗肿瘤效果。因此，联合乳酸代谢抑制剂的顺铂治疗方案，有望成为改善NSCLC患者化疗耐药的重要策略之一。

免疫治疗在NSCLC中已取得显著疗效，但由于肿瘤异质性，临床应用中仍不可避免地出现原发性和继发性耐药问题。乳酸转运体MCT1/MCT4抑制剂对具有耐药性的结肠癌和小细胞肺癌的免疫治疗具有逆转作用，可增强CD8^+ ^T细胞活性，抑制Tregs活性，逆转TAM极化，延缓肿瘤生长和转移^[50]^；一项NSCLC体内研究^[73]^中，草氨酸盐通过抑制LDHA活性，从而增强PD-1拮抗剂帕博利珠单抗的治疗效果。此外，GPR81和PD-1/PD-L1通路双重阻断会增强抗肿瘤作用^[74]^。LDHA抑制剂Sirpiglenastat可用于Kelch样ECH关联蛋白1/核因子红细胞2相关因子2/丝氨酸/苏氨酸激酶11（Kelch-like ECH-associated protein 1/nuclear factor, erythroid 2 like 2/serine/threonine kinase 11, *KEAP1*/*NFE2L2*/*STK11*）突变的晚期NSCLC患者，联合PD-L1抑制剂Atezolizumab表现出良好耐受性和初步疗效^[70]^。

## 5 小结与展望

代谢重编程是目前癌症研究中的热点，研究证实乳酸代谢在NSCLC进展中发挥重要作用。NSCLC细胞即使在有氧条件下仍偏好有氧糖酵解，产生大量乳酸。乳酸积累导致TME酸化，促进肿瘤细胞生存、增殖和转移，并通过多重机制推动NSCLC恶性进展。乳酸经MCT1、MCT4等MCTs外排，形成局部酸化TME，抑制CD8⁺ T细胞、NK细胞及DC的抗肿瘤活性，同时促进Tregs、MDSC和TAM等免疫抑制细胞扩增。乳酸可通过GPR81/HIF-1α通路上调PD-L1表达，降低免疫检查点抑制剂的疗效。多项研究已证实，乳酸不再是单纯的代谢终产物，还是重要的免疫调控分子，也是TME重塑的中心节点。靶向乳酸代谢的新药如LDHA抑制剂FX11、Sirpiglenastat及MCT1抑制剂AZD3965、MCT4抑制剂AZD0095已进入临床试验，体现了与免疫治疗协同增效的潜力。干预乳酸代谢有望改善TME，增强免疫治疗敏感性，推动NSCLC精准治疗。未来应聚焦乳酸相关代谢酶、转运体及其调控的免疫通路，推动“代谢-免疫”联合策略临床转化，为NSCLC患者提供更多治疗选择和生存获益。
